# The convergence epidemic volatility index (cEVI) as an alternative early warning tool for identifying waves in an epidemic

**DOI:** 10.1016/j.idm.2023.05.001

**Published:** 2023-05-07

**Authors:** Konstantinos Pateras, Eleftherios Meletis, Matthew Denwood, Paolo Eusebi, Polychronis Kostoulas

**Affiliations:** a Department of Public and One Health, School of Medicine, University of Thessaly, Karditsa, Terma Mavromichali St., 43131, Greece; b Department of Data Science and Biostatistics, University of Utrecht, Postbus 85500, 3508, GA, Utrecht, the Netherlands; c Department of Veterinary and Animal Sciences, University of Copenhagen, Grønnegårdsvej 8, 1870, Frederiksberg, Copenhagen, Denmark; d Department of Medicine and Surgery, University of Perugia, Via Gambuli, 1, 06132, Perugia, Italy

**Keywords:** Epidemic index, Surveillance system, Convergence diagnostics, Time-series, Early warning

## Abstract

This manuscript introduces the convergence Epidemic Volatility Index (cEVI), a modification of the recently introduced Epidemic Volatility Index (EVI), as an early warning tool for emerging epidemic waves. cEVI has a similar architectural structure as EVI, but with an optimization process inspired by a Geweke diagnostic-type test. Our approach triggers an early warning based on a comparison of the most recently available window of data samples and a window based on the previous time frame. Application of cEVI to data from the COVID-19 pandemic data revealed steady performance in predicting early, intermediate epidemic waves and retaining a warning during an epidemic wave. Furthermore, we present two basic combinations of EVI and cEVI: (1) their disjunction cEVI + that respectively identifies waves earlier than the original index, (2) their conjunction cEVI- that results in higher accuracy. Combination of multiple warning systems could potentially create a surveillance umbrella that would result in early implementation of optimal outbreak interventions.

## 1 Introduction

Intervention strategies that control the spread of an epidemic are required to sustain health, social and economic stability in times of local or global health crisis. Such strategies can be supported by early warning tools/systems that provide timely indication and adoption of preventive measures. Early warning systems focus on one or many of the following aspects; 1) modeling of the epidemics’ seasonality, 2) identification of the link between meteorological parameters and pathogens (Heffernan et al., 2004) and/or 3) spotting of spatial and temporal abnormalities in the expected number of cases (Vega et al., 2013; Yoneoka et al., 2021).

A number of methods already exist to monitor and identify initial and intermediate waves of an epidemic, such as the moving epidemic method that focuses on the start of the epidemic, growth models that provide predictions regarding the outbreak, methods based on machine learning algorithms that utilize associated parameters to indicate future epidemic waves (Chang et al., 2015) and the recently introduced epidemic volatility index (EVI), which is based on the already accumulated data and provides early warnings for both initial and intermediate epidemic waves (Kostoulas et al., 2021). The latter has been shown to provide accurate early warnings for COVID-19 epidemic data from a number of different countries including each individual state in the United States (Kostoulas et al., 2021). However, during the initial wave, as well as in special cases where sudden intermediate waves were observed, the original EVI algorithm is sometimes slow to identify an outbreak, which can result in delayed warnings.

EVI can be used in cases of novel emerging threats – like COVID-19 – where the disease has not been well studied, thus providing an ”early warning” relative to waiting for more traditional surveillance systems to adjust to the novel pathogen. Therefore, early warnings, as discussed in this manuscript, are not derived from predictive models (Alabdulrazzaq et al., 2021; Benvenuto et al., 2020; Proverbio et al., 2022). After the disease status is established, such early-warnings can be compared to “on-time detection” methods (Vega et al., 2013) while they differ from early warning signal (EWS) approaches that produce forecasts, e.g., by applying dynamic systems (Brett et al., 2017; Brett & Rohani, 2020; Southall et al., 2021).

In this manuscript we introduce the convergence Epidemic Volatility Index (cEVI) as a stand-alone alternative and as complementary methods (cEVI+, cEVI-) to the stand-alone EVI. EVI is briefly introduced, then, cEVI is presented and the differences to EVI are highlighted. An example application is given based on COVID-19 cases of four countries, namely; France, India, South Africa and the United States. We demonstrate that cEVI is a valid choice either as an alternative or as a complementary index to EVI, especially when the identification of rapid changes in intermediate epidemic waves is of importance.

## 2 Material and methods

### 2.1 The original epidemic volatility index - EVI

The original index (EVI) is based on the calculation of a rolling standard deviation for a series of data, for example, the number of new cases per day. At each algorithm's step and for a specific rolling window, cases within a window are obtained by shifting the window forward and calculating a new standard deviation. EVI is then calculated as the relative change between two consecutive rolling windows of size m. A warning signal is issued if the relative change exceeds a threshold (c) between zero and one, and simultaneously the observed cases at time (t) are higher than the previous weeks' reported cases average. Accuracy of EVI is measured by its sensitivity (Se) and Specificity (Sp) of the procedure (the probability of correctly issuing an early warning vs. the probability of correctly not signaling an early warning). These values are calculated in relation to a case definition, i.e., a percentage in the rise of mean number of cases between two consecutive weeks. An inner optimization algorithm based on the Youden's Index (J=Se+Sp−1), utilizes all sensitivities and specificities and then selects an optimal rolling window size (m’) and threshold (c’) for each time point (t) (Kostoulas et al., 2021). Based on the optimized combination of a window (m’) and a threshold (c’) an early warning is issued for time (t).

### 2.2 The convergence epidemic volatility index - cEVI

**Fig. 1:**
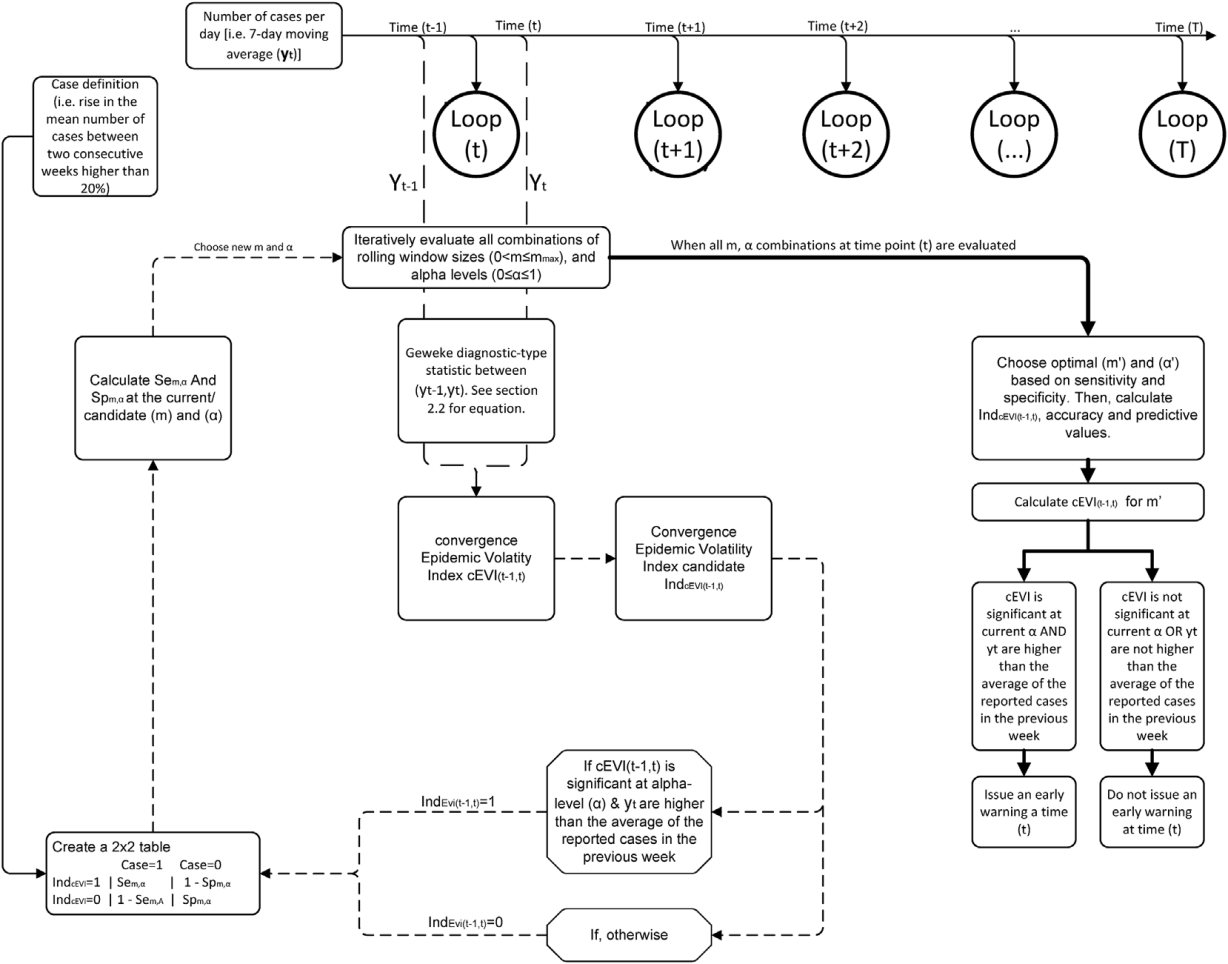
The convergence Epidemic Volatility Index (EVI) model presented as a graphical outline. T denotes the end of the time series while (t) denotes a time point of the time-series, Se and Sp stand for the sensitivity and specificity of the inner algorithm’s testing procedure calculated at each step of the algorithm. Solid lines are explanatory; dashed lines represent the iterative optimization algorithm at each time point (t) while the bold solid line denotes the end of the inner algorithm and the calculation of the final cEVI for time point (t).

The convergence epidemic volatility index, (cEVI) is based on the original EVI (Kostoulas et al., 2021) with an optimization algorithm inspired and led by a Geweke diagnostic-type statistic (Geweke, 1992), as observed in Fig. 1 and in Appendix (B. Model description, step 1). cEVI is calculated based on two consecutive windows of total size m resulting in an early window (m/2) and a late window (m/2). Based on these two non-overlapping but consecutive windows, two averages and standard deviations are calculated. At each step of the algorithm, these averages change as both windows are shifted forward one observation at a time. cEVI is then calculated as

$$cEVI_{t}=\frac{{\bar{y}}_{\left(t-m/2+1\right):t}-{\bar{y}}_{\left(t-m+1\right):\left(t-m/2\right)}}{\sqrt{2s_{\left(t-m/2+1\right):t}^{2}/m+2s_{\left(t-m+1\right):\left(t-m/2\right)}^{2}/m}}>t_{\alpha ,\left(m/2\right)-1}$$

An early warning is issued if the calculated value results in a statistically significant difference at a specific α level, while the observed cases at exact time (t) are higher than the previous weeks' reported cases average. As in EVI, accuracy of cEVI is measured by its Sensitivity and Specificity of the procedure. A similar criterion to EVI is applied to confirm true positive and true negative signals (i.e., a 20% percentage in the rise of mean number of cases between two consecutive weeks is considered), while again an optimization algorithm based on the Youden's Index (J=Se+Sp−1) selects an optimal total window size (m’) and instead of an optimal threshold (c’), it now optimizes the α-level for accepting a false negative test result. Based on the optimized combination of a window size (m’) and a (α’-level), a final early warning is issued for time (t). An overview of the procedure used to calculate cEVI is given in Fig. 1, and more specific details can be found in the online Appendix [B. Model description].

### 2.3 On combining multiple epidemic volatility indexes

EVI and cEVI can be applied through the EVI package (Meletis et al., 2022) via the deviant function. cEVI can directly be combined and/or compared with EVI via the graphical function evirlap. Conjunctions, (cEVI-: both EVI and cEVI must produce a signal), and/or disjunctions, (cEVI+: either EVI or cEVI produce a signal), of the stand-alone EVI and cEVI indices can be calculated and plotted. The aforementioned functions have been incorporated in the GitHub repository (https://github.com/ku-awdc/EVI) of EVI and they will be incorporated in the published EVI R package (Meletis et al., 2022, p. 7). All models and model comparisons have been developed in R (R Core Team, 2022).

## 3 Results

### 3.1 Motivating examples

**Fig. 2:**
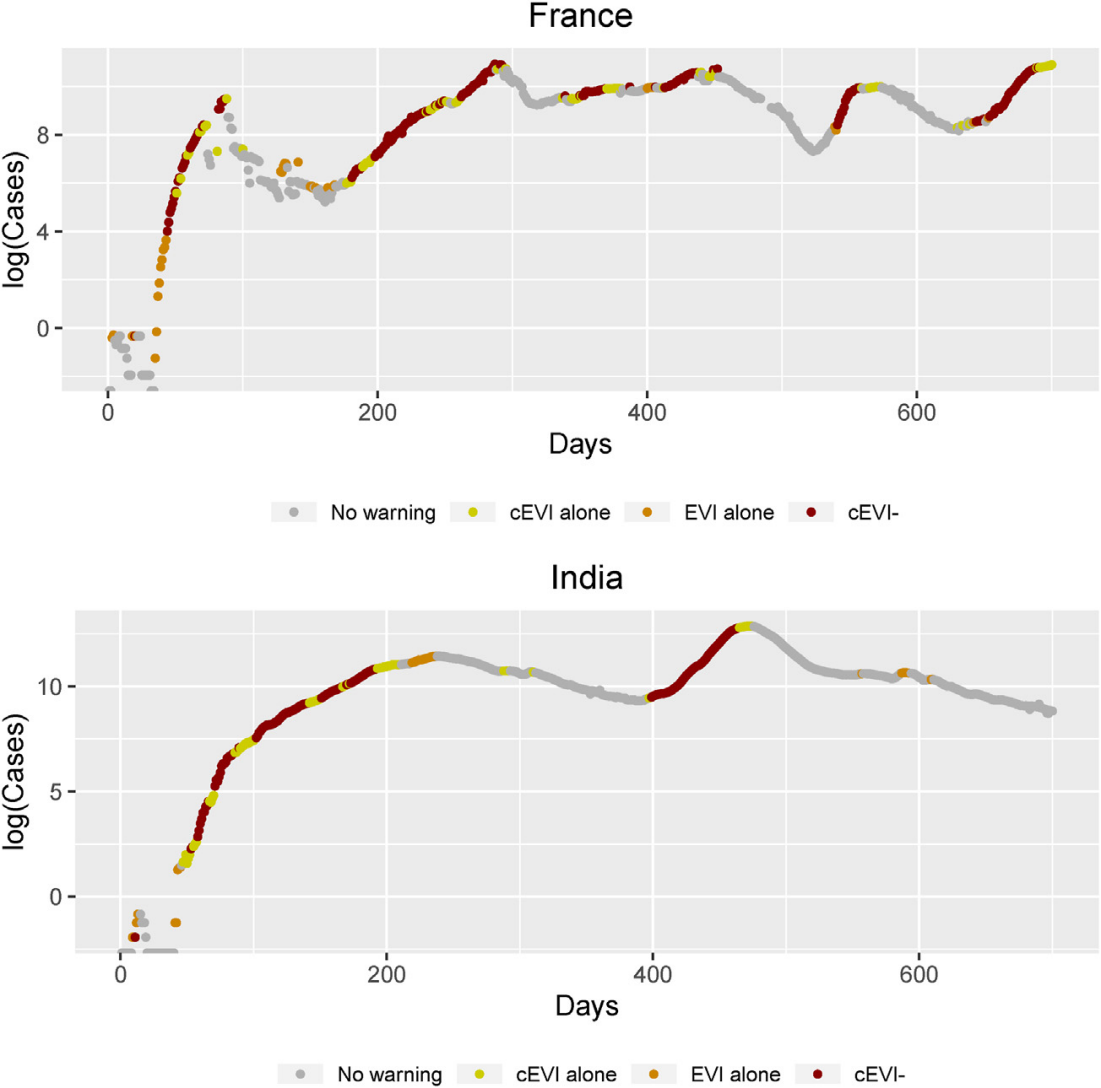
Combined early warnings for France and India on the logarithm of the moving average number of cases based on cEVI, EVI, cEVI+, cEVI- for the first 700 days. cEVI- is plotted as the conjunction of EVI and cEVI. All colored dots in each panel construct cEVI+, the disjunction of EVI or cEVI. EVI alone and cEVI- construct the EVI warnings, while cEVI alone and cEVI- construct the cEVI warnings. EVI alone and cEVI alone are warnings produced only by EVI or only by cEVI, respectively. The gray dots correspond to no warnings. Primary interest lies in early identifying an epidemic within each country individually and not to compare epidemics across countries that possibly have discrepancies in reporting and diagnostic testing. Alternative versions of Fig. 2 can be found in the Appendix where the total N days are shown for each country (Figs. A1, A2).

**Fig. 3:**
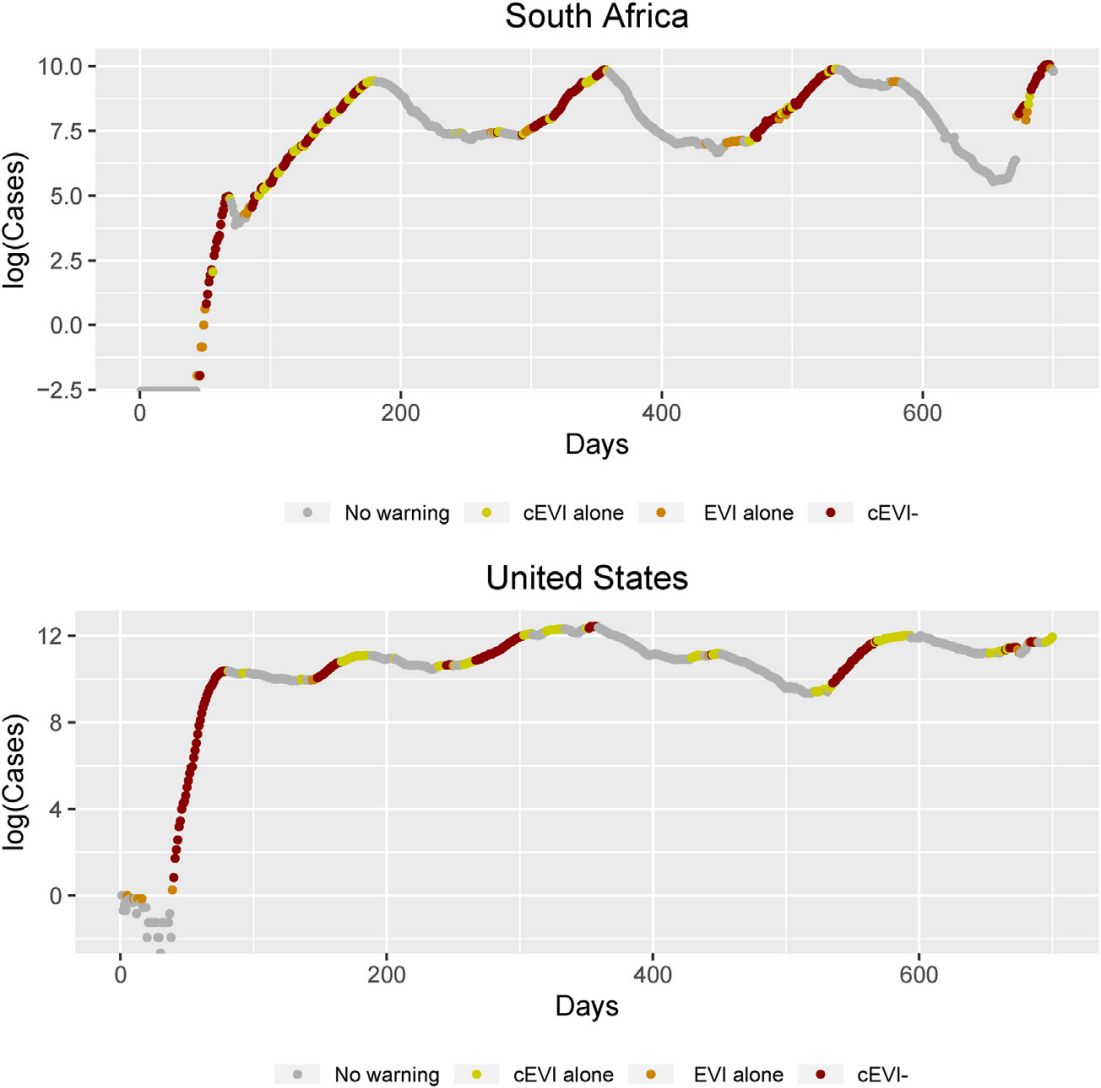
Combined early warnings for South Africa and the United States on the logarithm of the moving average number of cases based on cEVI, EVI, cEVI+, cEVI- for the first 700 days. cEVI- is plotted as the conjunction of EVI and cEVI. All colored dots in each panel construct cEVI+, the disjunction of EVI or cEVI. EVI alone and cEVI- construct the EVI warnings, while cEVI alone and cEVI- construct the cEVI warnings. EVI alone and cEVI alone are warnings produced only by EVI or only by cEVI, respectively. The gray dots correspond to no warnings. Primary interest lies in early identifying an epidemic within each country individually and not to compare epidemics across countries that possibly have discrepancies in reporting and diagnostic testing. Alternative versions of Fig. 3 can be found in the Appendix where the total N days are shown for each country (Figs. A1, A2).

Data on the four countries consist of the number of daily reported cases of COVID-19 up to March 9^th^ 2023. For demonstration purposes of cEVI, four countries were chosen with diverge wave characteristics, such as number of epidemic waves, peak intensity, time to main waves, and/or length of waves, as well as being on different continents. Similarly to the original EVI publication (Kostoulas et al., 2021), we focus on the reported confirmed cases, thus, we do not correct for discrepancies in the surveillance systems or other factors. Daily cases in South Africa consists of five waves, cases from France show two large late waves, cases of United States show smaller intermediate waves along with a single large wave, while cases in India consists of 2 very intense waves (Figs. A1, A2, Fig. 2, Fig. 3). These data can be retrieved from the COVID-19 Data Repository which is retained by the Center for Systems Science and Engineering (CSSE) at Johns Hopkins University (Dong et al., 2020). To minimize the non-biological variability of daily cases, similarly to EVI, a 7-day moving average has been calculated and provided to cEVI as input (in the same way as for EVI).

### 3.2 Analysis of examples

The ability of cEVI+, as a disjunction (i.e. identified by EVI or cEVI), to identify early new and intermediate waves can be seen in all presented countries and throughout most countries’ epidemic waves (Fig. 2, Fig. 3, Figs. A1, A2). Moreover, the ability of cEVI to continuously issue warnings during each wave seems more sufficient compared to EVI. cEVI demonstrates a more sensitive ability to provide daily warnings in comparison to EVI (Fig. 2, Fig. 3, Figs. A1, A2). Thus, it has the ability to early identify waves and retain a warning for an ongoing wave.

We further provide an empirical comparison across all 4 indices and a benchmark, the Farrigton's algorithm (Farrington et al., 1996), which was widely applied before the pandemic. In Table A1 (Appendix), interested readers can observe all 5 indices' relative behavior under 3 case definitions, an increase of 10%, 20% (original EVI) and 40% in cases between two consecutive weeks. To induce a more fair comparison we explored multiple α-levels for the Farrigton's algorithm. Across all 3 case definition scenarios and 4 countries, a) cEVI- index produced the most number of highest accuracies; b) cEVI+ produced the most number of highest negative predictive values; and c) both cEVI- and Farrigton's algorithm produced the most number of highest positive predictive values with small discrepancies in only a few of those scenarios (Table A1).

## 4 Discussion

As a disjunction of EVI or cEVI, cEVI + identifies and detects earlier COVID-19 epidemic's new and intermediate waves than either EVI or cEVI (Fig. 2, Fig. 3). This characteristic was tested in data from other countries and results remain consistent. The combined index provides indications of an upcoming epidemic wave earlier than the stand-alone indices (Fig. 2, Fig. 3). Absence of a common indication across EVI and cEVI, the cEVI- index, retained relative high accuracy and positive predictive values (Table A1), and it can therefore be suggestive of whether an early warning should be issued at all.

cEVI has been constructed under the same framework as EVI, although, this index handles optimization through different parameters; 1. the size of each window (m) and 2. the type I error level (α) are selected at each time point to optimize the Youden's index (Fig. 1). cEVI depends on more computational steps than EVI and even though both EVI and cEVI depend on relative simple computation, the inner optimization algorithm (dotted arrows in Fig. 1) combined with a large time series can make cEVI less computationally efficient. cEVI requires 1.5 times more time than EVI to be computed for a time series of 1-year time points. A function to update, only with new cases, the already run EVI and cEVI algorithms exists in the EVI R package deviant_update (Meletis et al., 2022). cEVI seems to identify better secondary epidemic waves and retain a warning, although its performance worsens during the peak of waves resulting in additional false positive results. Indeed, the positive and negative predictive values of the indices are impacted by the case definition and thus, their relative performance on identifying and retaining a warning, as observed in the empirical comparison of EVI approaches to the Farrigton's algorithm (Table A1). We should note that the applied original Farrigton's algorithm has been further extended and alternatives may perform better when data are available on local outbreaks (Yoneoka et al., 2021).

For future work, a more extensive (simulative/empirical) comparison of EVI, cEVI, their combinations and additional approaches could be undertaken (Brett et al., 2017; Southall et al., 2021; Yoneoka et al., 2021). This would require an objective early warning case definition by the construction of an empirical distribution for the case definition, i.e., the empirical distribution of the time difference between the first COVID-19 case for each country compared to the time of first lockdown. Furthermore, in this study, when defining the true COVID-19 cases per country, we did not account for discrepancies in surveillance systems between countries, discrepancies in types of diagnostic testing within and across countries (Pateras & Kostoulas, 2022) or discrepancies in national factors. As our main goal is to evaluate the performance of cEVI on identifying epidemic waves on surveillance data, we do not expect such discrepancies to impact the relative performance of cEVI to EVI. Radical changes in reporting of surveillance data by national authorities that create an influx of cases may signal an early warning for cEVI but it would certainly be identified as a false positive by each country.

The combined cEVI can be applied in the context of public health registry surveillance. Disjunctions (cEVI+) or conjunctions (cEVI-) of the stand-alone early warning indices can lead to further insights. cEVI+ can quickly identify temporal and spatial discrepancies, resulting in a valuable tool for policymakers, healthcare officials and any health-related organization that is interested in identifying abnormalities in an influx of health-related data, for instance, medicinal prescriptions, drug usage and/or exams prescriptions (Andreu-Perez et al., 2015; Reformed, 2021). cEVI- could also provide an early-warning tool for such systems with high accuracy rates with minimal data as input. Finally, we should also note that it is also important to consider the issue of colored correlations in signals, complex patterns and relationships within the time series data, to report a more informative underlying pattern. Thus, future early warning or on-time detection systems should also account for time-dependent covariates as factors of so-called dark figure (and other surveillance biases) of infections (Lazer et al., 2014).

## 5 Conclusion

Application of cEVI to real data from COVID-19 showed a consistently good performance in predicting early, intermediate epidemic waves while retaining a warning during an epidemic wave. The combined index, cEVI+, was shown to identify waves earlier than the original stand-alone EVI, while cEVI- demonstrated the ability to retain higher accuracy than the stand-alone EVI. Combination of multiple alarm systems has the potential to identify waves in a more efficient way and result in early – and thus optimal - implementation of interventions to control outbreaks.

## Funding

This work was funded by 10.13039/501100000921COST Action CA18208: HARMONY—Novel tools for test evaluation and disease prevalence estimation (https://harmony-net.eu/).

## Data and computing code

The data and the computing code are already available for replication in GitHub (https://github.com/ku-awdc/EVI) and they will become formally available in the already published R package EVI upon publication of the manuscript.

## Contribution analytical

Konstantinos Pateras: Conceptualization; Data curation; Formal analysis; Investigation; Methodology; Project administration; Software; Validation; Visualization; Writing - original draft.

Eleftherios Meletis: Validation; Software; Writing - review & editing.

Matthew Denwood: Validation; Software; Writing - review & editing.

Paolo Eusebi: Validation; Writing - review & editing.

Polychronis Kostoulas: Supervision; Investigation; Writing - review & editing.

## Declaration of competing interest

None.
