# Supplementary material for: The convergence epidemic volatility index (cEVI) as an alternative early warning tool for identifying waves in an epidemic
Source: Infect Dis Model. 2023 May 7;8(2):484–90. doi: 10.1016/j.idm.2023.05.001 (PMC10206801; doi:10.1016/j.idm.2023.05.001)
Supplement: Multimedia component 1 [file mmc1.docx]

**Online appendix
A. Figures**


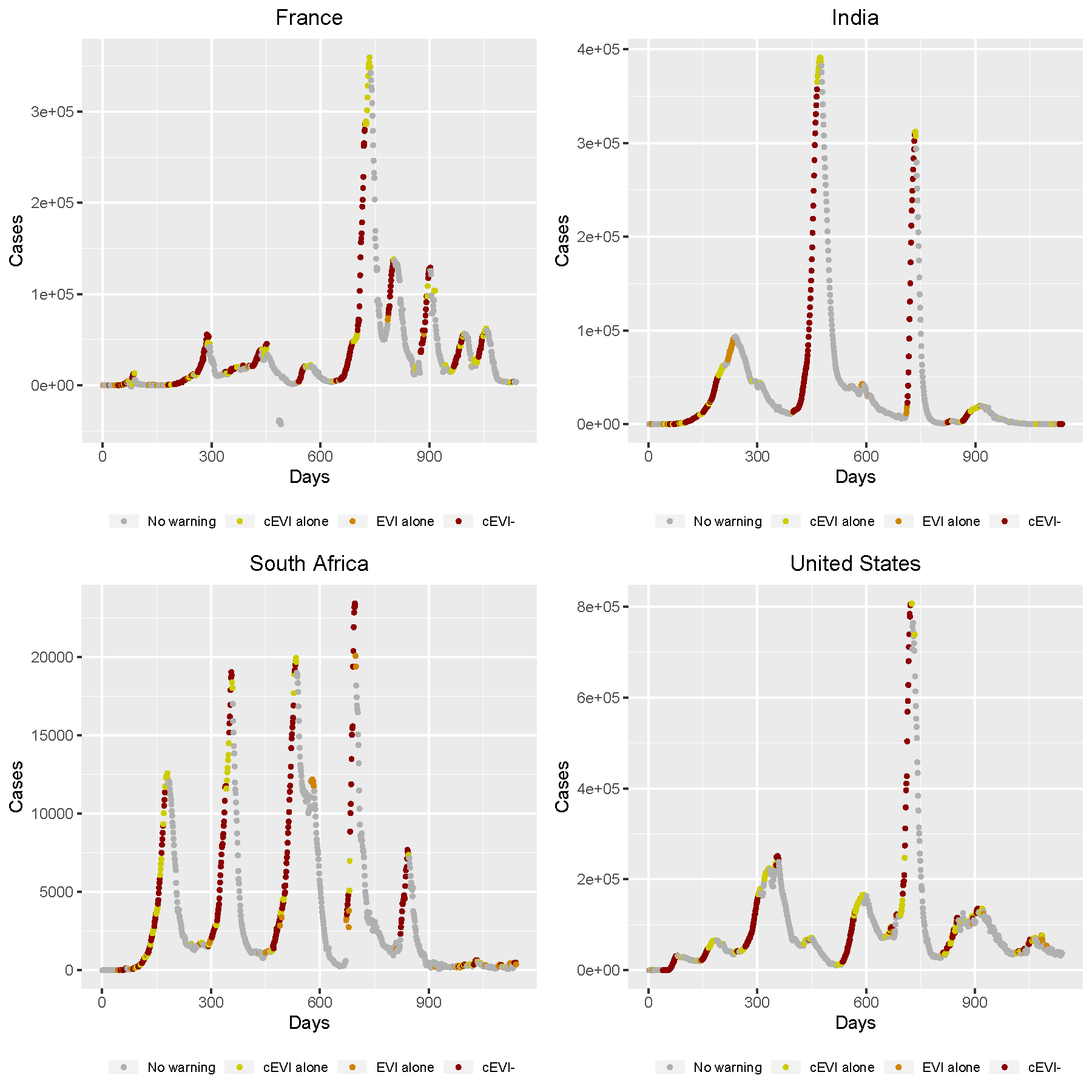


Figure A1. Combined early warnings on the moving average number of cases based on cEVI, EVI, cEVI+, cEVI- for the first 1140 days. cEVI- is plotted as the conjunction of EVI and cEVI. All colored dots in each panel construct cEVI+, the disjunction of EVI or cEVI. EVI alone and cEVI- construct the EVI warnings, while cEVI alone and cEVI- construct the cEVI warnings. EVI alone and cEVI alone are warnings produced only by EVI or only by cEVI, respectively. The gray dots correspond to no warnings.


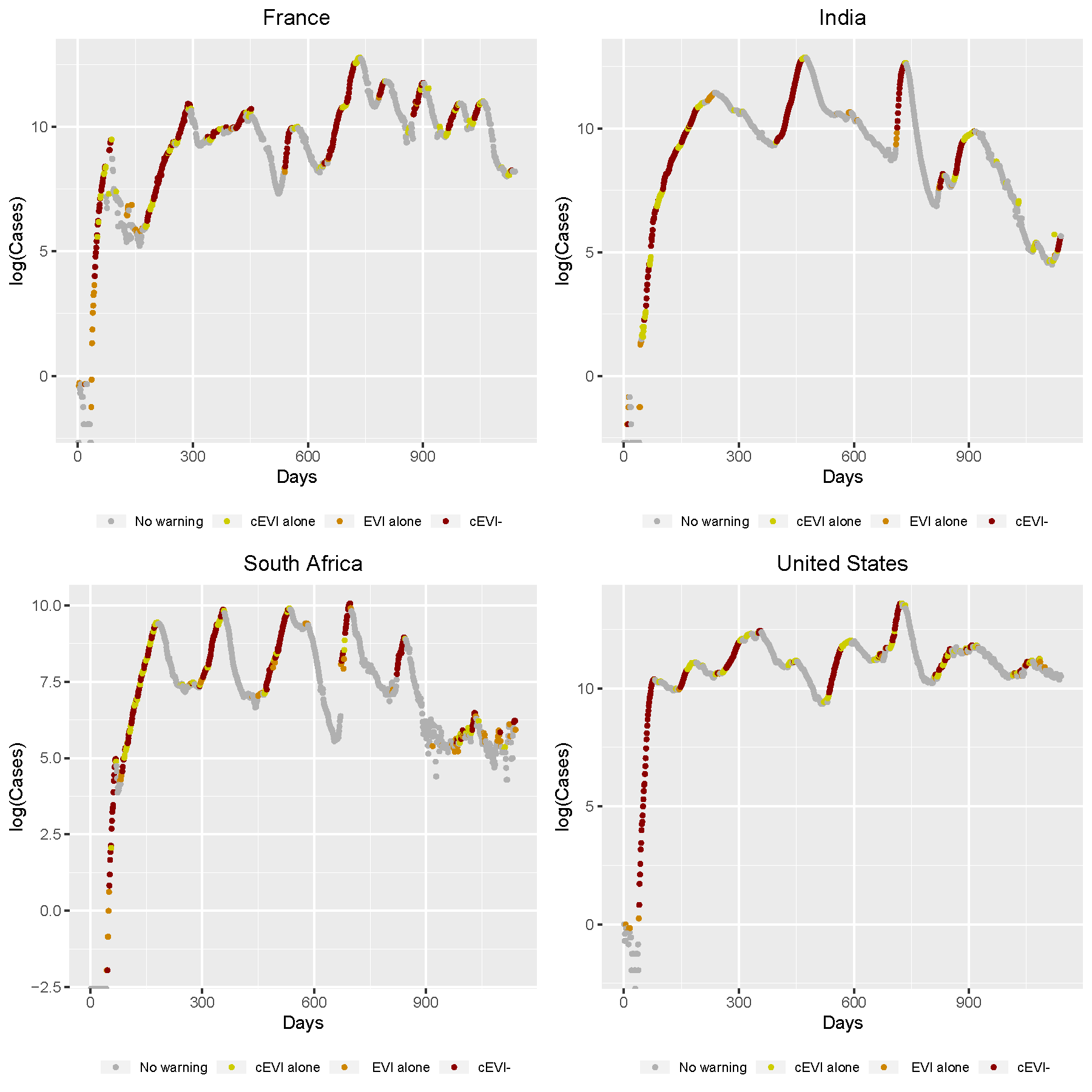


Figure A2. Combined early warnings on the logarithm of the moving average number of cases based on cEVI, EVI, cEVI+, cEVI- for the first 1140 days. cEVI- is plotted as the conjunction of EVI and cEVI. All colored dots in each panel construct cEVI+, the disjunction of EVI or cEVI. EVI alone and cEVI- construct the EVI warnings, while cEVI alone and cEVI- construct the cEVI warnings. EVI alone and cEVI alone are warnings produced only by EVI or only by cEVI, respectively. The gray dots correspond to no warnings.

**B. Model description**

Let $Y_{i}=(Y_{1},Y_{2},...,Y_{n})$ be a time series of length $N$, with two exclusive sequent windows of size $m/2$, ($m$ takes odd values), $m_{min}<m\leq m_{max}$, $m_{min}<m_{max}\leq N$, $t=N-m+1$ dyad of windows. Currently, $m_{min}=4$. The maximum chosen window size (m_max_) was set equal to 30 days, as in EVI, in order to give new data a chance to impact the convergence epidemic index and to aid the predictive ability of cEVI for intermediate epidemic waves.

At each step t

1. $cEVI_{t}=\frac{\overline{Y}_{(t-m+1):(t-m/2)}-\overline{Y}_{(t-m/2+1):t}}{\sqrt{\frac{2s_{(t-m+1):(t-m/2)}^{2}}{m}+\frac{2s_{(t-m/2+1):t}^{2}}{m}}}$
2. Set $Ind_{cEVI_{t-1,t}}$
   1. equal to 0 if $cEVI_{t}\leq t_{\alpha,m-1}$ or $Y_{t}\leq\overline{\mu_{t:t-7}}$,
   2. equal to 1, otherwise

where $t_{\alpha,m-1}$ denotes a critical value of the t distribution with *df=m-1*.

1. Calculate Se and Sp, up until time $t$, based on a criterion i.e. if positive cEVIs appear when a positive case definition really exists, as a percentage in the rise of mean number of cases between two consecutive weeks.
2. Optimize via $J=Se+Sp-1$ for all $m$ and $\alpha$ (instead of $c$) combinations for time $t$ and calculate (positive and negative predictive values) $PV_{t+},PV_{t-},Se,Sp$ for $t$. Based on the optimal total window size and $\alpha$-level issue a final warning for time $t$ based on similar criteria as in step 2.

**C. Empirical comparison**

| Country | Case definition | Algorithm | TN | FN | FP | TP | NPV | PPV | Se | Sp | Acc |
| --- | --- | --- | --- | --- | --- | --- | --- | --- | --- | --- | --- |
| France | 10 | Farrigton's | 628 | 450 | 10 | 51 | 58.3 | **98.4** | 0.1 | 0.98 | 59.6 |
| N=1136 |  | EVI | 541 | 234 | 95 | 266 | 69.8 | 85.1 | 0.53 | 0.85 | 71 |
|  |  | cEVI | 470 | 177 | 166 | 324 | 72.6 | 73.9 | 0.65 | 0.74 | 69.8 |
|  |  | cEVI+ | 448 | 155 | 188 | 345 | **74.3** | 70.4 | 0.69 | 0.7 | 69.8 |
|  |  | cEVI- | 563 | 255 | 73 | 245 | 68.8 | 88.5 | 0.49 | 0.89 | **71.1** |
|  | 20 | Farrigton's | 741 | 337 | 13 | 48 | 68.7 | **98.3** | 0.12 | 0.98 | 69.3 |
|  |  | EVI | 616 | 159 | 136 | 225 | 79.5 | 81.9 | 0.59 | 0.82 | 74 |
|  |  | cEVI | 526 | 121 | 226 | 264 | 81.3 | 69.9 | 0.69 | 0.7 | 69.5 |
|  |  | cEVI+ | 501 | 102 | 251 | 282 | **83.1** | 66.6 | 0.73 | 0.67 | 68.9 |
|  |  | cEVI- | 641 | 177 | 111 | 207 | 78.4 | 85.2 | 0.54 | 0.85 | **74.6** |
|  | 40 | Farrigton's | 886 | 192 | 25 | 36 | 82.2 | **97.3** | 0.16 | 0.97 | **80.9** |
|  |  | EVI | 678 | 97 | 231 | 130 | 87.5 | 74.6 | 0.57 | 0.75 | 71.1 |
|  |  | cEVI | 565 | 82 | 344 | 146 | 87.3 | 62.2 | 0.64 | 0.62 | 62.5 |
|  |  | cEVI+ | 537 | 66 | 372 | 161 | **89.1** | 59.1 | 0.71 | 0.59 | 61.4 |
|  |  | cEVI- | 706 | 112 | 203 | 115 | 86.3 | 77.7 | 0.51 | 0.78 | 72.3 |
| India | 10 | Farrigton's | 692 | 322 | 35 | 90 | 68.2 | 95.2 | 0.22 | 0.95 | 68.7 |
| N=1139 |  | EVI | 685 | 169 | 40 | 243 | 80.2 | 94.5 | 0.59 | 0.94 | 81.6 |
|  |  | cEVI | 631 | 128 | 94 | 284 | 83.1 | 87 | 0.69 | 0.87 | 80.5 |
|  |  | cEVI+ | 609 | 101 | 116 | 311 | **85.8** | 84 | 0.75 | 0.84 | 80.9 |
|  |  | cEVI- | 707 | 196 | 18 | 216 | 78.3 | **97.5** | 0.52 | 0.98 | **81.2** |
|  | 20 | Farrigton's | 768 | 246 | 48 | 77 | 75.7 | 94.1 | 0.24 | 0.94 | 74.2 |
|  |  | EVI | 724 | 130 | 90 | 193 | 84.8 | 88.9 | 0.6 | 0.89 | 80.7 |
|  |  | cEVI | 666 | 93 | 148 | 230 | 87.7 | 81.8 | 0.71 | 0.82 | 78.8 |
|  |  | cEVI+ | 631 | 79 | 183 | 244 | **88.9** | 77.5 | 0.76 | 0.78 | 77 |
|  |  | cEVI- | 759 | 144 | 55 | 179 | 84.1 | **93.2** | 0.55 | 0.93 | **82.5** |
|  | 40 | Farrigton's | 869 | 145 | 76 | 49 | 85.7 | **92** | 0.25 | 0.92 | **80.6** |
|  |  | EVI | 769 | 85 | 174 | 109 | 90 | 81.5 | 0.56 | 0.82 | 77.2 |
|  |  | cEVI | 694 | 65 | 249 | 129 | 91.4 | 73.6 | 0.66 | 0.74 | 72.4 |
|  |  | cEVI+ | 657 | 53 | 286 | 141 | **92.5** | 69.7 | 0.73 | 0.7 | 70.2 |
|  |  | cEVI- | 806 | 97 | 137 | 97 | 89.3 | 85.5 | 0.5 | 0.85 | 79.4 |
| South | 10 | Farrigton's | 640 | 375 | 44 | 80 | 63.1 | 93.6 | 0.18 | 0.94 | 63.2 |
| Africa |  | EVI | 590 | 234 | 92 | 221 | 71.6 | 86.5 | 0.49 | 0.87 | 71.3 |
| N=1139 |  | cEVI | 602 | 195 | 80 | 260 | 75.5 | 88.3 | 0.57 | 0.88 | **75.8** |
|  |  | cEVI+ | 553 | 163 | 129 | 292 | **77.2** | 81.1 | 0.64 | 0.81 | 74.3 |
|  |  | cEVI- | 639 | 266 | 43 | 189 | 70.6 | **93.7** | 0.42 | 0.94 | 72.8 |
|  | 20 | Farrigton's | 692 | 323 | 53 | 71 | 68.2 | **92.9** | 0.18 | 0.93 | 67 |
|  |  | EVI | 629 | 195 | 114 | 199 | 76.3 | 84.7 | 0.51 | 0.85 | 72.8 |
|  |  | cEVI | 639 | 158 | 104 | 236 | 80.2 | 86 | 0.6 | 0.86 | **77** |
|  |  | cEVI+ | 587 | 129 | 156 | 265 | **82** | 79 | 0.67 | 0.79 | 74.9 |
|  |  | cEVI- | 681 | 224 | 62 | 170 | 75.2 | 91.7 | 0.43 | 0.92 | 74.8 |
|  | 40 | Farrigton's | 821 | 194 | 73 | 51 | 80.9 | **91.8** | 0.21 | 0.92 | **76.6** |
|  |  | EVI | 691 | 133 | 201 | 112 | 83.9 | 77.5 | 0.46 | 0.77 | 70.6 |
|  |  | cEVI | 682 | 115 | 210 | 130 | 85.6 | 76.5 | 0.53 | 0.76 | 71.4 |
|  |  | cEVI+ | 620 | 96 | 272 | 149 | **86.6** | 69.5 | 0.61 | 0.7 | 67.6 |
|  |  | cEVI- | 753 | 152 | 139 | 93 | 83.2 | 84.4 | 0.38 | 0.84 | 74.4 |
| United | 10 | Farrigton's | 660 | 265 | 127 | 87 | 71.4 | 83.9 | 0.25 | 0.84 | 65.6 |
| States |  | EVI | 698 | 181 | 87 | 171 | 79.4 | 88.9 | 0.49 | 0.89 | 76.4 |
| N=1137 |  | cEVI | 579 | 134 | 206 | 218 | 81.2 | 73.8 | 0.62 | 0.74 | 70.1 |
|  |  | cEVI+ | 558 | 120 | 227 | 232 | **82.3** | 71.1 | 0.66 | 0.71 | 69.5 |
|  |  | cEVI- | 719 | 195 | 66 | 157 | 78.7 | **91.6** | 0.45 | 0.92 | **77** |
|  | 20 | Farrigton's | 762 | 163 | 158 | 56 | 82.4 | 82.8 | 0.26 | 0.83 | 71.8 |
|  |  | EVI | 790 | 89 | 128 | 130 | 89.9 | 86.1 | 0.59 | 0.86 | 80.9 |
|  |  | cEVI | 641 | 72 | 277 | 147 | 89.9 | 69.8 | 0.67 | 0.7 | 69.3 |
|  |  | cEVI+ | 616 | 62 | 302 | 157 | **90.9** | 67.1 | 0.72 | 0.67 | 68 |
|  |  | cEVI- | 815 | 99 | 103 | 120 | 89.2 | **88.8** | 0.55 | 0.89 | **82.2** |
|  | 40 | Farrigton's | 836 | 89 | 178 | 36 | 90.4 | 82.4 | 0.29 | 0.82 | 76.6 |
|  |  | EVI | 834 | 45 | 178 | 80 | 94.9 | 82.4 | 0.64 | 0.82 | 80.4 |
|  |  | cEVI | 681 | 32 | 331 | 93 | **95.5** | 67.3 | 0.74 | 0.67 | 68.1 |
|  |  | cEVI+ | 651 | 27 | 361 | 98 | 96 | 64.3 | 0.78 | 0.64 | 65.9 |
|  |  | cEVI- | 864 | 50 | 148 | 75 | 94.5 | **85.4** | 0.6 | 0.85 | **82.6** |

Table A1. Empirical comparison of EVI, cEVI, cEVI+, cEVI- and Farrigton’s algorithms across the four studied countries up until 9^th^ March 2023 (France, India, South Africa and the United States). With bold the largest value of each algorithm is highlighted per scenario for the Positive predictive value (PPV), Negative predictive value (NPV) and the total Accuracy (Acc) of the algorithms. The sensitivity (Se) and specificity Sp) is also provided. TN, FP, FN, TP refer to the number of true/false positives/negatives based on the assumed case definition. The values refer to a total of N time points, however the relative performance of each method remain relatively similar for earlier time points.
